# Editorial: Neuroeconomy and Neuromarketing: The Study of the Consumer Behaviour in the COVID-19 Context

**DOI:** 10.3389/fpsyg.2022.822856

**Published:** 2022-03-15

**Authors:** Juan Jose Blazquez-Resino, Santiago Gutierrez-Broncano, Edyta Gołąb-Andrzejak

**Affiliations:** ^1^Faculty of Social Sciences, Department of Business Administration, University of Castilla-La Mancha, Ciudad Real, Spain; ^2^Faculty of Management and Economics, Department of Marketing, Gdańsk University of Technology, Gdańsk, Poland

**Keywords:** neuroeconomy, neuromarketing, consumer, behavior, COVID-19

From 2020, the world must face a health emergency caused by SARS-CoV-2, the effects of which have been compared to natural disasters (De Vito and Gomez, 2020; Sarkis et al., 2020). The crisis caused by COVID-19 has brought profound changes in all aspects of society and its economic, social, political and cultural repercussions are expected to show in the coming years. This poses a major challenge to societies. The spread of COVID-19 globally, caused government authorities in almost all countries to start taking drastic measures, such as limiting the free movement of people and placing restrictions on most activities (Donthu and Gustafsson, 2020).

As Sheth (2020) states, consumption and consumer behavior is highly dependent on the market context. In this sense, this unprecedented situation has had a profound impact on consumer habits and attitudes (Donthu and Gustafsson, 2020; Pantano et al., 2020). Some works have tried to study the immediate effects of the pandemic on consumer behavior (e.g., Kirk and Rifkin, 2020; Laato et al., 2020; Pantano et al., 2020; Sheth, 2020), among which the hoarding of products, the development of new forms of consumption, based on improvisation and creativity or the increase in consumption through electronic channels or streaming, stand out. In short, consumers have learned to improvise, adapt and modify their consumption motivations. However, the long-term implications of this pandemic are yet to be determined. The effects of COVID-19 are also expected to have long-lasting effects on consumer habits and behaviors. In particular, confinement has meant that the emotional sphere of consumers has been affected (Piccarozzi et al., 2021), and many of the behaviors developed in the coming years will, in many cases, be based on subconscious motivations.

Taking advantage of advances related to 4.0 and 5.0 technologies, consumer behavior is beginning to be studied from a neuroscientific perspective. The knowledge of the most emotional and subconscious aspects of the consumer allows us to analyze the processes of choice and decision in a more complete way. From this perspective, it will be important to enhance the study of Neuromarketing and neuroeconomics, which involves the study of brain responses that explain the behavior and decision making of people, in the face of certain sensory and experiential stimuli from the outside (Braidot, 2014). Recent advances in technology have made it possible to use brain imaging in organizational settings at relatively low cost and in a practical way to further neuroscience research efforts in business settings (Waldman et al., 2019). Although the brain changes, and each brain is different, the intention is to predict some behaviors common to all humans (Patiño and Goldáraz, 2014) through the use of different techniques.

Therefore, to address the study of consumer behavior in the post-COVID-19 era, the present Research Topic brings together a set of papers that attempt to study how different factors triggered by the pandemic have achieved a significant effect on consumers' behavioral intentions. These papers examine different subtopics related to food, health products, collaborative economy and, of course, neuroscience. Globally, the objectives of this special issue try to broadly reach the different perspectives in the study of consumer behavior in the context of COVID-19, considering:

- A study of the effects of COVID-19 on consumer behavior in the short term.
- An analysis of the economic effects of COVID-19.
- The social and psychological changes brought about by the current situation of social change.
- A study of consumer behavior from the perspective of neuromarketing and/or neuroeconomics.
- Forecasting of changes in habits and behaviors caused by COVID-19.
- The development of new marketing and distribution systems aimed at meeting new market needs.
- New retailing techniques adapted to consumer changes.

To achieve these objectives, first, the work of Balconi and Sansone provides a broad understanding of the relationship between neuroscience and consumer behavior. The authors present a state of the art review of consumer neuroscience, which clearly shows the evolution in the development of the different works on the use of neurophysiological tools. The rest of the published papers develop scientific studies with the aim of showing some of the main markets where the pandemic has had significant effects, such as food, product hoarding and collaborative economies.

Specifically, Liu and Zhen's work focuses on the study of direct and indirect relationships between food safety incidents and organic food purchase, demonstrating a direct and significant relationship on the improvement of consumer organic cognition. This work has important implications for the marketing process of sustainable and organic products. For their part, Cannito et al. attempt to study the effects of temporary discounts during the COVID-19 pandemic as a function of the level of hoarding shown by the consumer. The authors show an increased consumer preference for a key health product during the pandemic, surgical masks. Overall, the study identifies the role of the general level of hoarding, contributing to the theoretical debate on impulsivity in hoarder behavior during times of crisis. Finally, the article by Garaus and Garaus shows the effects of safety claims and environmental claims on the intention to use carsharing. This work serves to identify the mental processes that guide consumer decision making during the coronavirus crisis and offers an important insight into this.

We encourage readers to read all the articles included in this special issue and try to draw their own conclusions about the complex phenomena of consumer behavior in a post-COVID-19 future.

## Author Contributions

All authors listed have made a substantial, direct, and intellectual contribution to the work and approved it for publication.

## Conflict of Interest

The authors declare that the research was conducted in the absence of any commercial or financial relationships that could be construed as a potential conflict of interest.

## Publisher's Note

All claims expressed in this article are solely those of the authors and do not necessarily represent those of their affiliated organizations, or those of the publisher, the editors and the reviewers. Any product that may be evaluated in this article, or claim that may be made by its manufacturer, is not guaranteed or endorsed by the publisher.
